# Investigating the necessity of bilateral common femoral vein ultrasound in patients with unilateral symptomatic deep venous thrombosis

**DOI:** 10.1016/j.jvsv.2025.102242

**Published:** 2025-04-01

**Authors:** Moira A. McGevna, Molly Ratner, Giancarlo Speranza, Keerthi B. Harish, Mikel Sadek, Glenn R. Jacobowitz, Karan Garg, Thomas S. Maldonado, Caron B. Rockman

**Affiliations:** a Rutgers Robert Wood Johnson Medical School, Piscataway, NJ; b Division of Vascular Surgery, New York University Langone Health, New York, NY; c Department of Vascular Surgery, Hackensack University Medical Center, Hackensack, NJ

**Keywords:** Deep venous thrombosis, Duplex venous ultrasound, Imaging, Chronic venous changes, Superficial venous thrombosis

## Abstract

**Objective:**

Venous duplex ultrasound (VDUS) examinationis the accepted initial imaging study to rule out lower extremity deep venous thrombosis (DVT). In accordance with the Intersocietal Accreditation Commission vascular laboratory policies, many institutions require technicians to additionally assess the asymptomatic contralateral common femoral vein (CFV). There is conflicting literature on whether this policy is needed. Therefore, the aim of this study was to investigate the utility of examining the asymptomatic contralateral CFV in patients undergoing a unilateral lower extremity VDUS to rule out DVT by (1) defining the prevalence of DVT in the contralateral asymptomatic limb and (2) identifying risk factors that predispose patients to develop a DVT in the asymptomatic limb.

**Methods:**

We retrospectively reviewed the results of all unilateral lower extremity VDUS examinations performed on inpatients and outpatients from January 2023 to July 2023. Patient data, including age, sex, symptoms, risk factors for DVT, and indications for the study, were collected. The primary outcome was the frequency of DVT in the asymptomatic contralateral CFV. Categorical and continuous data were compared using the χ^2^ and Student *t* tests, respectively. For all tests, a *P* value of less than .05 was considered statistically significant.

**Results:**

We identified 371 patients (170 inpatient and 201 outpatient) with unilateral DVT symptoms who underwent VDUS examination during the study period. Right leg symptoms were present in 186 patients (50%) and left leg symptoms were present in 185 patients (50%). The overall incidence of acute DVT in the symptomatic limb was 17% (17.4% outpatient vs 16.5% inpatient; *P* = .NS). Outpatients were more likely to have superficial venous thrombosis (7.0% vs 0.6%; *P* = .002) and chronic venous changes (25.4% vs 1.2%; *P* < .001) in the symptomatic limb. Of the DVTs in the symptomatic limb, 59% were documented in the calf veins, 25% in the proximal veins, and 16% in both the proximal and calf veins. There were no incidences of bilateral DVT in our cohort. Moreover, none of the patients had a DVT isolated to the contralateral CFV.

**Conclusions:**

Scanning the asymptomatic contralateral CFV may not be necessary for patients undergoing unilateral VDUS examination for symptomatic DVT, regardless of thrombotic risk factors. A single-extremity study suffices in most cases; if implemented, this strategy will improve vascular laboratory efficiency and decrease costs without a decrease in DVT detection.

**Article Highlights**

• **Type of Research:** Single-center retrospective cohort study
• **Key Findings:** In patients with unilateral symptomatic deep venous thrombosis (DVT), we observed no thrombus occurrence in the contralateral common femoral vein.
• **Take Home Message:** Patients with unilateral symptomatic DVT may not need a venous duplex ultrasound examination in the contralateral common femoral vein. A single-extremity study will suffice in most cases and, if implemented, will improve vascular laboratory efficiency without a decline in DVT detection.

Nine hundred thousand people in the United States are diagnosed with a deep venous thrombosis (DVT) per year.^1^ If left untreated, 1 in 10 people with a DVT will develop a pulmonary embolism (PE), a potentially life-threatening complication.^2^ Therefore, a quick and accurate diagnosis of DVT is needed. Venous duplex ultrasound (VDUS) tests are accepted as the initial imaging study to rule out lower extremity DVT in patients with suggestive symptoms such as pain and swelling. VDUS tests are noninvasive and have a high degree of sensitivity and specificity.^3^

To maintain the accuracy of imaging tests like VDUS, the Intersocietal Accreditation Commission (IAC) accredits and provides testing standards and performance criteria for noninvasive imaging at vascular laboratories.^4^ To be accredited by the IAC, some vascular laboratories, including ours, are required to scan the asymptomatic contralateral common femoral veins (CFVs) in patients presenting with unilateral lower extremity DVT symptoms. The presumed rationale behind this recommendation is to ensure that there is no proximal DVT in the asymptomatic contralateral limb. To our knowledge and experience, it is extraordinarily rare to diagnose any abnormality in the asymptomatic contralateral CFV. Moreover, performing a bilateral VDUS when it is not indicated clinically decreases efficacy, increases costs, and negatively affects vascular laboratory workflow.

Thus, our study aimed to investigate the necessity and utility of scanning the contralateral CFV in symptomatic patients undergoing a unilateral lower extremity VDUS test to rule out DVT by (1) defining the prevalence of DVT or other abnormalities in the contralateral asymptomatic limb and (2) identifying risk factors to help identify the population predisposed to the development of DVT in the asymptomatic limb.

## Methods

We performed a single-center, retrospective analysis of all patients who underwent a unilateral lower extremity VDUS examination from January 2023 to July 2023. Data were collected for every patient using electronic medical records. Demographics, past medical history, thrombotic risk factors, and VDUS results were documented. VDUS examination was conducted on all patients at our accredited inpatient and outpatient laboratory with ultrasound technicians and performed according to a standard protocol. The scan was performed from the level of the groin to the ankle in all symptomatic extremities. In the asymptomatic extremity, VDUS was performed in the CFV only. Imaging of the proximal veins included the CFV, femoral, profunda femoris, and popliteal veins. Examination of the calf veins included the posterior tibial, peroneal, soleal, and gastrocnemius veins. On VDUS examination, an acute DVT was defined as a dilated, noncompressible vein lacking flow. Both occlusive and nonocclusive acute DVTs were included. In contrast, chronic venous change was defined as a small lumen vein that was partially compressible and contained multiple flow channels. Additionally, a bilateral CFV waveform analysis was completed on all patients to assess for proximal thrombus. Findings from each limb were compared and assessed for symmetry and phasicity. Symmetric and biphasic waveforms indicated an absence of thrombus. Further investigation of the proximal limb was conducted if the waveform was asymmetric and if there was a loss of phasicity or if there was a presence of a continuous waveform. DVT risk factors included prolonged immobility, which was defined as recent extended air travel, car travel, or hospitalization where the patient was bedbound within the prior 6 months. Patients were noted to have undergone recent surgery if it occurred within the prior 6 months, and documented to have an active malignancy if they received a diagnosis or to have had chemotherapy treatment within 1 year of their presentation. Obesity was defined as a patient having a body mass index of 30 or greater. A patient was classified as having unilateral signs and symptoms of DVT if they exhibited pain and swelling in one extremity. Patients with bilateral DVT symptoms and those for whom a bilateral VDUS examination was requested by the ordering physician were excluded from our analysis. We also excluded patients who were undergoing VDUS for postprocedural evaluation after an endovenous ablation procedure.

We compared DVT risk factors and outcomes between inpatient and outpatient cohorts. The primary outcome of our study was the frequency of DVT in the asymptomatic contralateral CFV. Secondary outcomes included examining demographics, past medical history, and thrombotic risk factors for patients undergoing VDUS examination to rule out DVT, as well as those with a confirmed DVT.

All statistical analyses were performed using SPSS (IBM Corp, Version 28, Armonk, NY). Categorical and continuous variables were calculated using Pearson χ^2^ and two-sample *t* test, respectively. Categorical data was reported as frequencies or percentages and continuous variables were reported as means ± standard deviations. For all tests, a *P* value of less than .05 was considered statistically significant.

Approval for this study was obtained from the New York University Institutional Review Board. The data were deidentified during collection, and patient consent was waived owing to the retrospective nature of the study. This study was conducted in accordance with the Health Insurance Portability and Accountability Acts and the prevailing ethical principles governing research.

## Results

We identified 371 patients during the study period (170 inpatients and 201 outpatients). Demographics, past medical history, and thrombotic risk factors are described in Table I. The median age was 62 years and the majority of our cohort was female (55.5%). DVT risk factors were present, including a prior history of DVT/PE (28.6%), varicose veins (27.2%), and recent surgery (15.1%). Table II demonstrates findings in the symptomatic limb between the inpatient and outpatient cohorts. Outpatients were more likely to have chronic venous changes (25.4% vs 1.2%; *P* < .001) and superficial venous thrombosis (7.0% vs 0.6%; *P* = .002) in the symptomatic limb. Seventeen percent of the total cohort was found to have an acute DVT in the symptomatic limb, specifically 16.5% in the inpatient and 17.4% in the outpatient setting (*P* = .81). The Fig illustrates the location of the thrombus in the symptomatic limb of patients with acute DVT. The majority of thrombi were found in the calf veins (59%), followed by the proximal veins (25%). Additionally, 16% of cases showed thrombus present in both the proximal and calf veins.

**Table I:** Demographics, past medical history, and deep vein thrombosis (*DVT*) risk factors in the overall cohort

Variables	All patients (n = 371) No. (%)	Inpatients (n = 170) No. (%)	Outpatients (n = 201) No. (%)	*P* value
Demographics				
Age, years	62 ± 9	62 ± 7	63 ± 10	.97
Female sex	206 (55.5)	95 (55.8)	111 (55.2)	.99
Thrombotic risk factors				
History of DVT/PE	106 (28.6)	22 (12.9)	84 (42.0)	**<.001**
Malignancy	21 (5.6)	13 (7.6)	8 (3.9)	.91
Surgery	56 (15.1)	29 (17.1)	27 (13.4)	.95
Trauma	37 (9.9)	22 (12.9)	15 (7.5)	.90
Immobility	42 (11.1)	26 (14.7)	16 (8.0)	.88
Varicose veins	101 (27.2)	21 (12.4)	80 (39.8)	**<.001**
Thrombophilia	21 (5.7)	5 (2.9)	16 (8.0)	**.04**
Pregnancy/postpartum	12 (3.2)	4 (2.4)	8 (3.9)	.38
Past medical history				
Coronary artery disease	57 (15.4)	35 (20.6)	22 (10.9)	**.01**
Cerebrovascular incident	22 (5.9)	12 (7.1)	10 (5.0)	.40
Obesity	107 (29.4)	57 (34.5)	50 (25.1)	.049
Current smoker	24 (6.5)	14 (8.2)	10 (5.0)	.20

*PE,* Pulmonary embolism.

Values are mean ± standard deviation or number (%).

Boldface *P* values represent statistical significance.

**Table II:** Venous duplex ultrasound (VDUS) findings in the symptomatic limb

Findings	All patients (n = 371) No. (%)	Inpatients (n = 170) No. (%)	Outpatients (n = 201) No. (%)	*P* value
Acute DVT	63 (17.0)	28 (16.5)	35 (17.4)	.81
Chronic venous changes	53 (14.3)	2 (1.2)	51 (25.4)	**<.001**
SVT	15 (4.0)	1 (0.6)	14 (7.0)	**.002**

*DVT,* Deep venous thrombosis; *SVT,* superficial venous thrombosis.

Boldface *P* values represent statistical significance.

**Fig:**
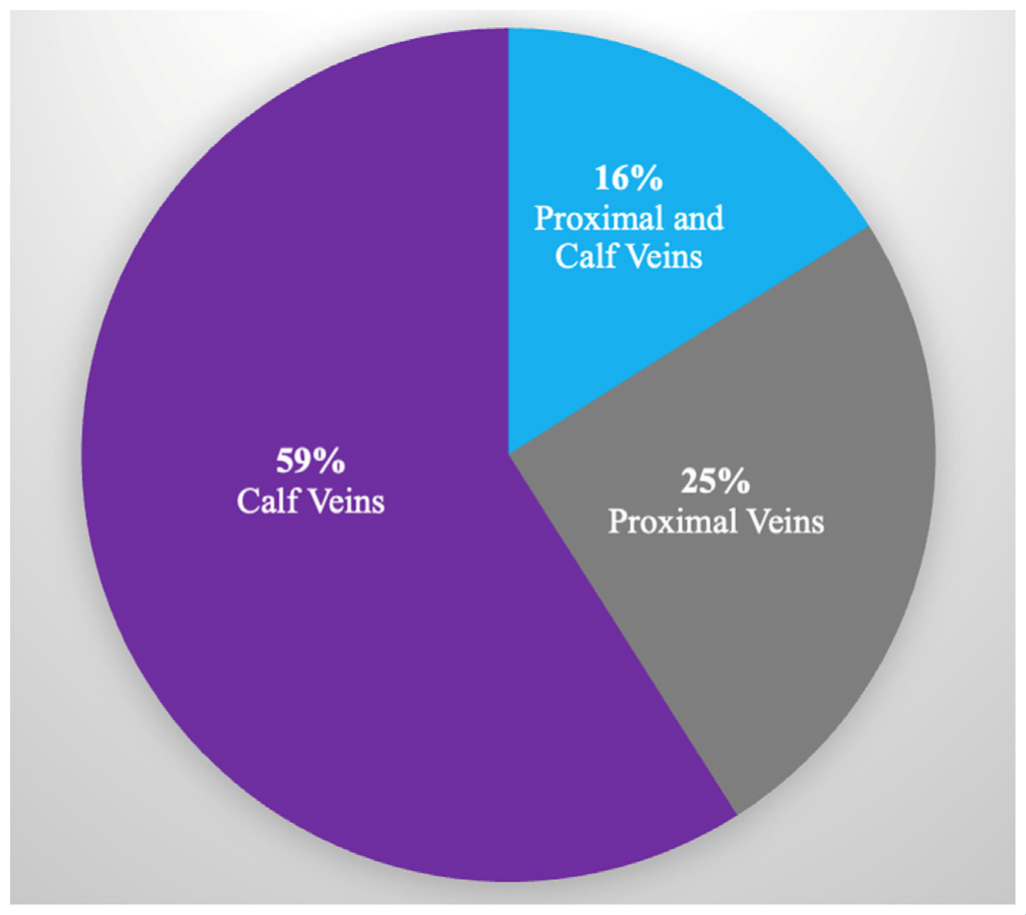
Acute deep vein thrombosis (DVT) in the symptomatic limb, thrombus location.

Table III presents the demographics, past medical history, and thrombotic risk factors of patients diagnosed with acute DVT in the symptomatic limb, updating our cohort to 63 patients. The patients in this cohort exhibited a higher proportion of DVT risk factors compared to the previous, such as a history of DVT/PE (35.5%), recent surgery (34.9%) or immobility (23.8%), and the presence of varicose veins (31.7%). Outpatients were more likely to have a history of varicose veins (42.9% vs 17.9%; *P* = .03), while inpatients were more likely to be current smokers (17.9% vs 2.9%; *P* = .04). Table IV summarizes VDUS findings in the asymptomatic CFV. No patients had an acute DVT, but one outpatient exhibited chronic venous changes. Furthermore, none of the patients in our cohort had an isolated clot in the asymptomatic contralateral CFV. Finally, all bilateral CFV waveforms demonstrated a phasic pattern with no indications of proximal thrombus.

**Table III:** Demographics, past medical history, and deep vein thrombosis (*DVT*) risk factors in patients with acute DVT in the symptomatic limb

	Acute DVT patients (n = 63) No. (%)	Inpatients (n = 28) No. (%)	Outpatients (n = 35) No. (%)	*P* value
Demographics				
Age, years	62 ± 9	61 ± 9	63 ± 8	.62
Female sex	30 (47.6)	12 (42.9)	18 (51.4)	.50
Thrombotic risk factors				
History of DVT/PE	17 (27.0)	11 (39.2)	6 (21.4)	.07
Malignancy	4 (6.3)	3 (10.7)	1 (2.9)	.20
Surgery	22 (34.9)	11 (39.3)	11 (31.4)	.52
Trauma	7 (11.1)	3 (10.7)	4 (11.4)	.93
Immobility	15 (23.8)	9 (32.1)	6 (17.1)	.17
Varicose veins	20 (31.7)	5 (17.9)	15 (42.9)	**.03**
Pregnancy/postpartum	2 (3.2)	1 (3.6)	1 (2.9)	.87
Hormonal therapy	8 (12.7)	2 (7.1)	6 (17.1)	.24
Thrombophilia	3 (4.8)	1 (3.6)	2 (5.7)	.69
Past medical history				
Coronary artery disease	5 (7.9)	3 (10.7)	2 (5.7)	.47
Current smoker	6 (9.5)	5 (17.9)	1 (2.9)	**.04**
Obesity	12 (20.7)	5 (20.0)	7 (21.2)	.91
Cerebrovascular incident	6 (9.5)	4 (14.3)	2 (5.7)	.25

*PE,* Pulmonary embolism.

Values are mean ± standard deviation or number (%).

Boldface *P* values represent statistical significance.

**Table IV:** Venous duplex ultrasound (VDUS) findings in the asymptomatic contralateral common femoral vein (CFV)

	Acute DVT patients (n = 63) No. (%)	Inpatients (n = 28) No. (%)	Outpatients (n = 35) No. (%)	*P* value
Acute DVT	0	0	0	NS
Chronic venous changes	1 (1.6)	0 (0.0)	1 (2.9)	.37
SVT	0	0	0	NS

*DVT,* Deep venous thrombosis; *NS*, not significant; *SVT,* superficial venous thrombosis.

## Discussion

This study aimed to evaluate the necessity of performing a VDUS in the asymptomatic, contralateral CFV of patients with unilateral DVT symptoms. We provided more recent data on this widely discussed topic, which has been debated for decades and still lacks a unified consensus. Previous research has included single-center studies that document varying rates of contralateral DVT occurrence, resulting in differing conclusions and recommendations. In this study, the positive acute DVT rate in the symptomatic limb was low, at 17%. We observed no differences in the incidence rates of acute DVT between the inpatient and outpatient settings, whether in symptomatic or asymptomatic limbs. Additionally, we did not identify any cases of clinically silent acute DVT in the contralateral CFV. Our findings suggest that bilateral imaging may not be necessary, even in patients with thrombotic risk factors. Other investigatots have agreed with this reasoning, claiming that there should strictly be imaging of the symptomatic limb only owing to the low rates of contralateral DVT.^5^^,^^6^ Moreover, they discovered that the presence of a DVT in the contralateral limb did not alter patient management. Thus, conducting a bilateral examination would likely increase costs and resource use with a very low clinically meaningful return on those resources.

However, previous authors have suggested that all patients should undergo imaging of both extremities owing to the common occurrence of bilateral DVT and the potential for unsuspected contralateral DVT.^7^ Pennell et al^8^ found a 34% incidence of asymptomatic contralateral thrombus and, therefore, recommended bilateral studies. Importantly, they found that patients in their cohort with clinically silent DVT, especially in the proximal veins, would not have been treated adequately if their anticoagulation therapy regimen was based solely on the findings from the symptomatic limb. They advocate that bilateral studies provides additional information that would be useful for the follow-up care of the patient. Some experts advocate for more patient-specific approaches, recommending bilateral imaging primarily for hospitalized patients or those with thrombotic risk factors, such as malignancy. They emphasize the importance of developing a patient-specific algorithm to determine whether a bilateral scan is warranted.^8^^,^^9^

In our cohort, we observed a high incidence of calf vein DVTs at 59%, compared with 25% in proximal veins alone. Our cohort differs from other studies, which have shown that most symptomatic patients have thrombus located in the proximal veins.^10^^,^^11^ The location of the DVT in the symptomatic limb has been found to correlate with the location of DVT in the asymptomatic limb. Sobreira et al^12^ identified a 92.6% correlation between proximal DVT on the symptomatic side and involvement in the asymptomatic limb. The femoropopliteal region was affected most frequently in both symptomatic cases and asymptomatic limbs. Only 3.7% of cases had a symptomatic distal DVT and an asymptomatic proximal DVT. The authors emphasized that patients with duplex findings of symptomatic DVT extending to the CFVs should have both limbs examined.^13^

Doppler waveform analysis is used commonly to assess proximal venous obstruction, with waveform distortions serving as a reliable indicator of thrombus presence.^14^ According to the IAC guidelines, bilateral CFV waveform analyses are required for patients undergoing VDUS to evaluate unilateral symptomatic DVT. However, our findings suggest that both compression and waveform analysis of the asymptomatic leg are unnecessary, particularly in patients with symptomatic calf DVT. This is because the incidence of contralateral proximal DVT, especially in the iliac veins, is low in these cases.

The low rate of DVT in the asymptomatic contralateral CFV in our cohort may be attributed to the fact that many DVTs in the symptomatic limb were located in the calf veins. Patients with symptomatic below-knee DVT are less likely to have asymptomatic proximal DVT in the contralateral leg. This finding indicates that VDUS exmination of the contralateral CFV may not be necessary for those patients. Therefore, conducting unilateral studies can save both time and costs without compromising the detection or management of DVTs.

There are several limitations to this study. Its retrospective nature and the use of a single center limits the generalizability of our findings. Each institution has its own protocols, and prior research has used various investigative methods. For example, some studies focus on asymptomatic patients, scan the entire contralateral leg, or include patients exclusively in either the inpatient or outpatient setting. At our institution, as outlined in our methods section, the ultrasound technician is required to scan the contralateral CFV as part of the assessment, in accordance with IAC recommendations. However, the IAC suggests that each laboratory have an individual protocol for unilateral examinations.^15^ Blebea et al^16^ documented the heterogeneity of protocols among different institutions and found that approximately 25% of accredited vascular laboratories conduct a bilateral VDUS examination in patients with unilateral symptoms. However, the specific contralateral veins that need to be assessed are not defined clearly or uniform across these laboratories.^16^ Therefore, the differences in contralateral DVT findings may be attributed to these variations. Using similar research methodologies in future studies is essential for achieving consensus and a deeper understanding of this topic. Last, only 69 patients were identified with acute DVT in the symptomatic limb, which significantly limited our sample size for further investigation of thrombus presence in the contralateral limb. Although no thrombus was detected in the contralateral limb within our cohort, the small number of patients with thrombus in the symptomatic limb means that even a small number of cases in the contralateral limb could alter the overall incidence rate substantially.

Future directions include conducting a meta-analysis to better identify patients who would benefit from a bilateral CFV ultrasound examinaton and to assess the feasibility of creating a risk analysis protocol for that decision. Additional research is required to assess patients who are incidentally found to have asymptomatic contralateral DVT, especially in the proximal veins, and to establish whether this is associated with significant morbidity, such as post-thrombotic syndrome. Finally, further exploration of cost implications should be pursued.

## Conclusions

Our work suggests that scanning the asymptomatic contralateral CFV may not be necessary for patients with unilateral symptomatic DVT, regardless of thrombotic risk factors. A single-extremity study will suffice in most cases, and, if implemented, it will improve vascular laboratory efficiency and decrease costs without a decrease in DVT detection.

## Author contributions

Conception and design: MM, CR

Analysis and interpretation: MM, MR, GS, KH, MS, GJ, KG, TM, CR

Data collection: MM

Writing the article: MM, MR, GS

Critical revision of the article: MM, MR, GS, KH, MS, GJ, KG, TM, CR

Final approval of the article: MM, MR, GS, KH, MS, GJ, KG, TM, CR

Statistical analysis: Not applicable

Obtained funding: Not applicable

Overall responsibility: CR

## Funding

None.

## Disclosures

None.
